# Supplementary figures and images for: Trends in social determinants of child health and perinatal outcomes in European countries 2005–2015 by level of austerity imposed by governments: a repeat cross-sectional analysis of routinely available data
Source: BMJ Open. 2018 Oct 12;8(10):e022932. doi: 10.1136/bmjopen-2018-022932 (PMC6194462; doi:10.1136/bmjopen-2018-022932)

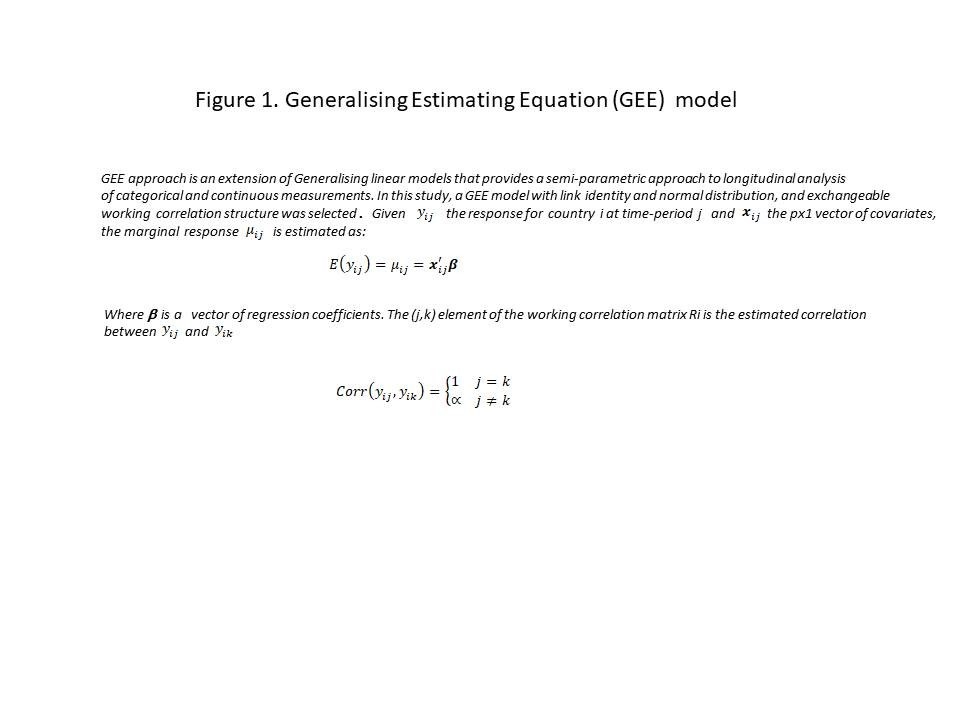

Supplement: Supplementary data [file bmjopen-2018-022932supp001.jpg]

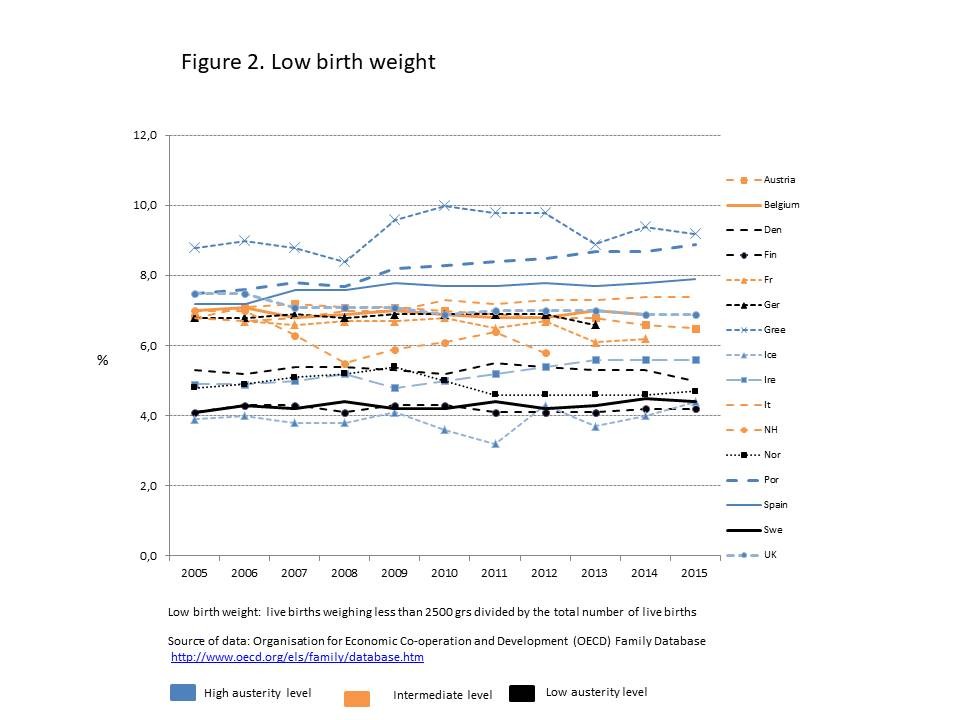

Supplement: Supplementary data [file bmjopen-2018-022932supp002.jpg]

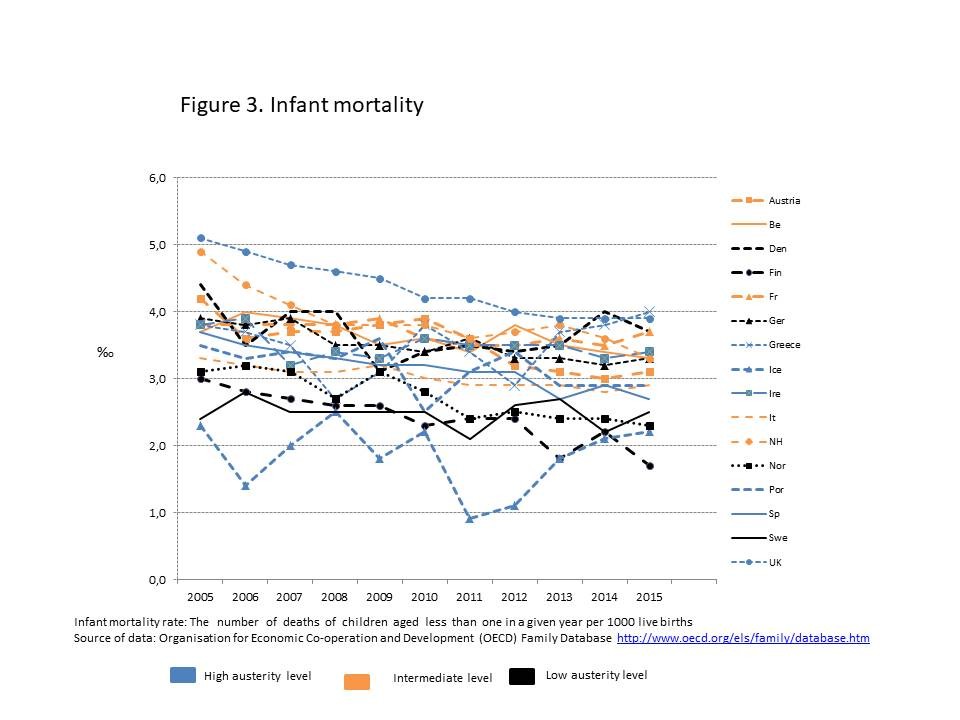

Supplement: Supplementary data [file bmjopen-2018-022932supp003.jpg]

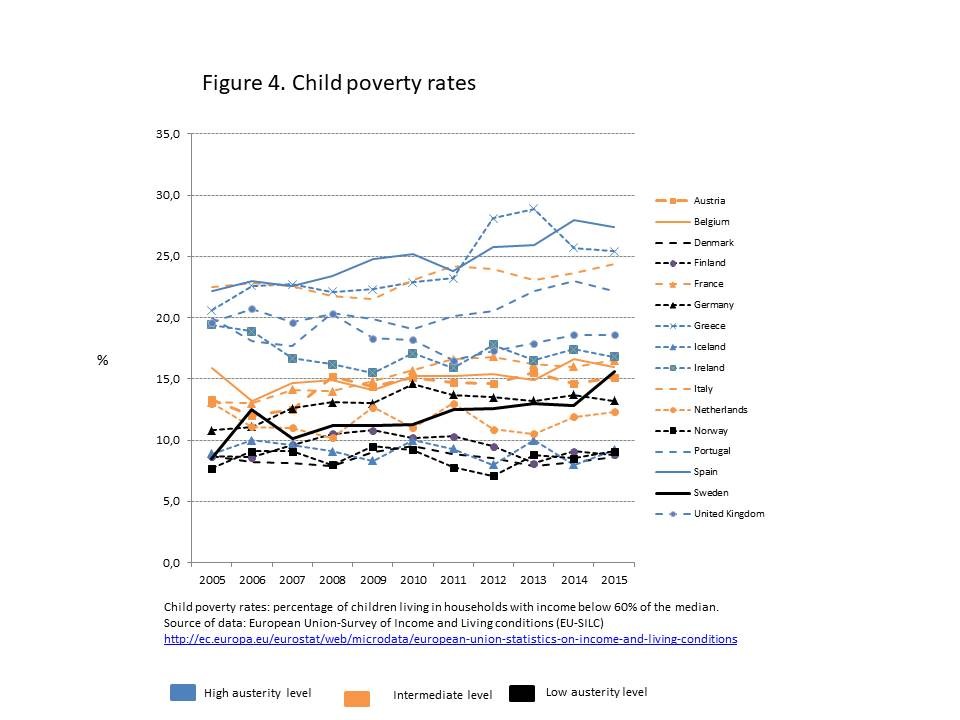

Supplement: Supplementary data [file bmjopen-2018-022932supp004.jpg]

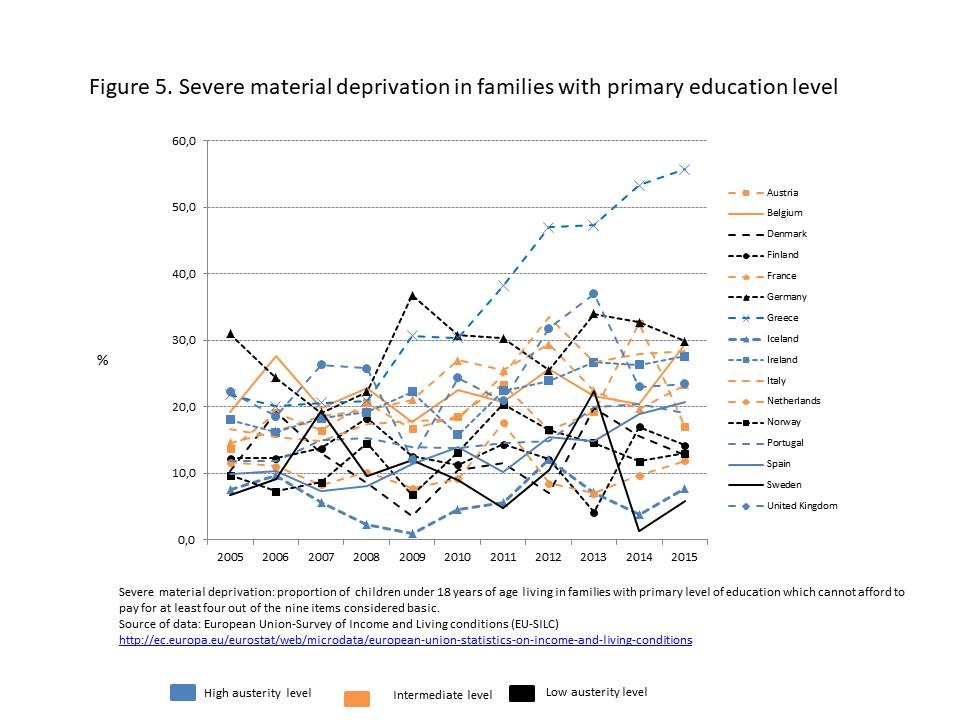

Supplement: Supplementary data [file bmjopen-2018-022932supp005.jpg]

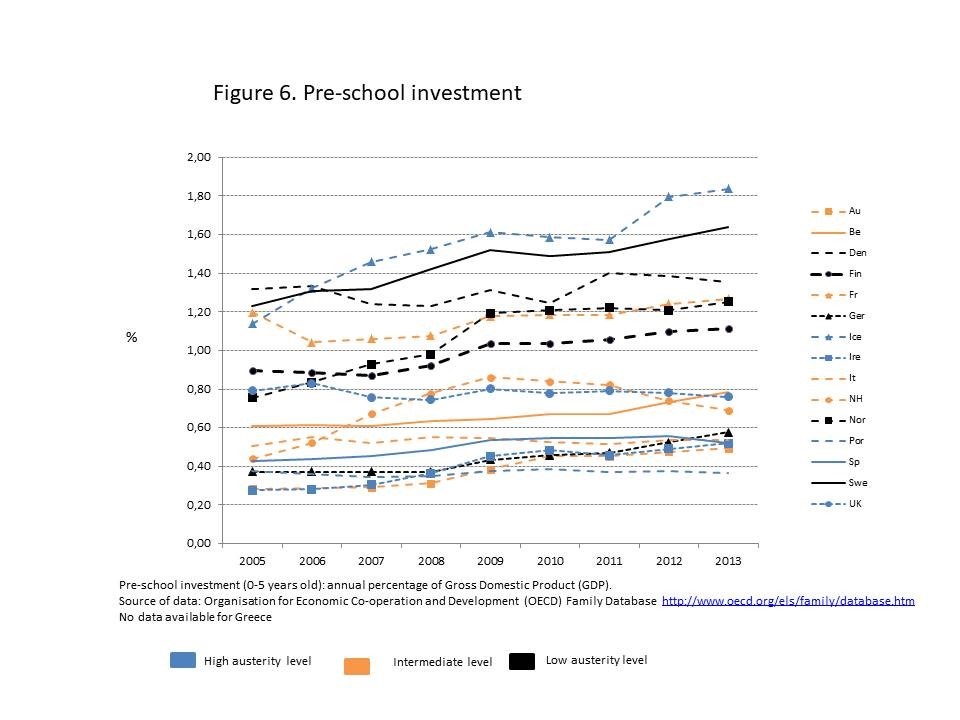

Supplement: Supplementary data [file bmjopen-2018-022932supp006.jpg]
