# Supplementary material for: Trends in social determinants of child health and perinatal outcomes in European countries 2005–2015 by level of austerity imposed by governments: a repeat cross-sectional analysis of routinely available data
Source: BMJ Open. 2018 Oct 12;8(10):e022932. doi: 10.1136/bmjopen-2018-022932 (PMC6194462; doi:10.1136/bmjopen-2018-022932)
Supplement: Author's manuscript [file bmjopen-2018-022932.draft_revisions.pdf]

BMJ Open

BMJ Open is committed to open peer review. As part of this commitment we make the peer review history of every article we publish publicly available.

When an article is published we post the peer reviewers' comments and the authors' responses online. We also post the versions of the paper that were used during peer review. These are the versions that the peer review comments apply to.

The versions of the paper that follow are the versions that were submitted during the peer review process. They are not the versions of record or the final published versions. They should not be cited or distributed as the published version of this manuscript.

BMJ Open is an open access journal and the full, final, typeset and author-corrected version of record of the manuscript is available on our site with no access controls, subscription charges or pay-per-view fees (http://bmjopen.bmj.com).

If you have any questions on BMJ Open's open peer review process please email info.bmjopen@bmj.com

BMJ Open

Trends in social determinants of child health and perinatal outcomes in rich countries 2005-2015 by level of austerity imposed by governments

Journal:	*BMJ Open*
Manuscript ID	bmjopen-2018-022932
Article Type:	Research
Date Submitted by the Author:	13-Mar-2018
Complete List of Authors:	Rajmil, Luis; Retired,<br>Taylor-Robinson, David; University of Liverpool,<br>Gunnlaugsson, Geir; Faculty of Social and Human Sciences University of Iceland<br>Hjern, Anders; Centre for Health Equity Studies (CHESS) Stockholm University/Karolinska Institutet<br>Spencer, Nick; University of Warwick, School of Health and Social Studies
Keywords:	austerity, child health, perinatal outcomes, social determinants of health

SCHOLARONE™
Manuscripts

For peer review only

Trends in social determinants of child health and perinatal outcomes in rich countries 2005-2015 by level of austerity imposed by governments

Authors:
Luis Rajmil,[1] David Taylor-Robinson,[2] Geir Gunnlaugsson,[3] Anders Hjern,[4] Nick Spencer[5]

[1] Retired, paediatrician and public health and epidemiology specialist. Homer 22 1rst 1 Barcelona Spain
[2] Clinical Senior Lecturer, Department of Public Health and Policy, Whelan Building University of Liverpool ,Liverpool, UK
[3] Faculty of Social and Human Sciences, University of Iceland, Reykjavik, Iceland
[4] Centre for Health Equity Studies (CHESS), Stockholm University/Karolinska Institutet, SE-106 91, Stockholm, Sweden
[5] Emeritus Professor of Child Health, Division of Mental Health and Wellbeing, Warwick Medical School University of Warwick, Coventry, CV4 9JD, UK

Corresponding author:
Luis Rajmil Homer 22, 1rst, 1. Barcelona 08023. Spain
E-mail: 12455lrr@comb.cat
Phone +34 93 4173243

For peer review only - http://bmjopen.bmj.com/site/about/guidelines.xhtml

Abstract

Objective To assess whether the level of austerity implemented by national governments was associated with adverse trends in perinatal outcomes and the social determinants of children's health (SDCH) in rich countries

Design Longitudinal ecological study of country level time trends in perinatal outcomes and SDCH and from 2005-2015

Setting and participants 16 European countries, plus Australia and Canada using available data from the International Monetary Fund (IMF), the Organisation for Economic Co-operation and Development (OECD), and Eurostat.

Main outcome measures Trends in perinatal outcomes (low birth weight (LBW); infant mortality (IM)) and the social determinants of child health (SDCH: child poverty rates; severe material deprivation in families with primary education; pre-school investment) in 3 time-periods: 2005-07, 2008-10 and 2012-15. Outcomes were compared according to the cyclically adjusted primary balance (CAPB, differences between 2013 and 2009) as a measure of austerity, stratified in tertiles. Generalised estimating equation (GEE) models of repeated measures were used to assess time-trend differences in 3 periods.

Results Countries with higher levels of austerity had worse outcomes, mainly at the last study period. Material deprivation increased during the period 2012-15 in those countries with higher CAPB (interaction CAPB-period 2012-15, B: 5.62: p< 0.001), as did LBW (interaction CAPB-period 2012-15, B: 5.27; p<0.001).

Conclusions Countries that implemented more severe austerity measures have experienced increasing material deprivation in families with primary education, and LBW, worsening the negative impact of economic crisis. Reversing austerity policies that impact children is likely to improve child health outcomes.

Words in the text: 2337
Words in the abstract: 245
References: 30
Tables: 5

For peer review only - http://bmjopen.bmj.com/site/about/guidelines.xhtml

Strengths and limitations of this study

- This is one of the first studies that allowing the analysis of trends in SDCH and perinatal indicators starting before the economic crisis.
- One of the main strengths of the study include the use of nationally representative data with standardised and comparable definitions and methods, and a study design that allowed us to analyse time trends starting in 2005, 3 years before the economic crisis, through to 2015.
- Countries included in the study may not allow the results to be generalized
- Variables used to assess austerity, SDCH, and perinatal outcomes may have limited ability to discriminate specific measures taken by Governments

For peer review only

For peer review only - http://bmjopen.bmj.com/site/about/guidelines.xhtml

Introduction

The global financial crisis starting in 2008 has had a great economic impact around the world, although the impact in each country depends on several factors, such as the starting point, mechanisms of social protection and social transfers, and the measures adopted by governments to deal with the crisis.[1] During the period 2008-10 the initial financial crisis was followed by a downturn in economic activity, resulting in evictions, foreclosures, and prolonged unemployment in many countries. From 2010-11 onward the pressure to adopt and enforce austerity measures, mainly within the European Union and worldwide, has been levied by global financial institutions such as the International Monetary Fund.[2-4] These austerity measures taken by governments were neither homogeneous nor similarly implemented in rich countries; some protected public sector programs and systems while others instituted large budget cuts in education, health and other public services.

Assessing the health effects of the crisis is challenging given the difficulty establishing association of exposure and outcomes. There is a lag time between exposure to the effects of a severe economic downturn and adverse health outcomes[5] and, in the case of exposure in early childhood, the adverse effects may not emerge until adulthood.[6] Childhood is an especially vulnerable period to the main determinants of health, from socioeconomic and environmental macro conditions to individual life style factors. All factors related to financial capital (i.e. family income), human capital (i.e. education), and social capital (family working conditions) have potential influence on future child health and development.[7,8] Moreover, there is overwhelming evidence for the profound effects of social factors on health throughout childhood and into adulthood.[9-11]

Findings from studies to date on the impact of the economic crisis on perinatal outcomes and social determinants of child health (SDCH), carried out at national and international level show foetal and childhood populations as population groups most affected by the crisis.[12-16] Vulnerability starts in the prenatal period with adverse effects on perinatal outcomes.[17-19] Most studies of the impact of economic crisis on health have not distinguished between economic crises themselves and policy responses to these crises.[6] Furthermore there is a paucity of literature on the impact of austerity measures imposed by government in response to the economic crisis on perinatal outcomes and SDCH.[20]

The aim of the study was therefore to assess time trends in perinatal outcomes and SDCH by level of austerity enforced by governments. We hypothesise that those countries that implemented and maintained high levels of austerity would show statistically significant differences in adverse trends in perinatal outcomes and SDCH compared with countries imposing lower levels of austerity.

For peer review only - http://bmjopen.bmj.com/site/about/guidelines.xhtml

Methods

We undertook a longitudinal ecological study of trends in perinatal outcomes and SDCH at country level, and assessed how these trends varied according to the level of austerity measures implemented. Sixteen European countries plus Australia and Canada were included in the study, using routinely available data for the period 2005-2015 pertaining to perinatal outcomes and SDCH.

Outcome variables

Perinatal outcomes: Data on perinatal outcomes were taken from the Organisation for Economic Co-operation and Development (OECD) Family Database (http://www.oecd.org/els/family/database.htm).

1. Low birth weight (LBW) was defined as the number of live births weighing less than 2500 grs divided by the total number of live births.
2. Infant mortality: deaths of children aged less than one year per 1000 live births (no minimum threshold of gestation period or birth weight).

Social determinants of child health (SDCH):

1. Child poverty was defined as the percentage of children living in households with income below 60% of the median. Data for European countries were taken from the European Union-Survey of Income and Living conditions (EU-SILC) (http://ec.europa.eu/eurostat/web/microdata/european-union-statistics-on-income-and-living-conditions) and for Australia and Canada from the OECD Family Database.
2. Severe material deprivation rate was defined as the proportion of children under 18 years of age living in families with primary level of education which cannot afford to pay for at least four out of the nine items considered basic, such as having arrears on mortgage or rent payments, utility bills, hire purchase instalments or other loan payments; not being able to afford one week's annual holiday away from home; not being able to afford a meal with meat, chicken, fish (or vegetarian equivalent) every second day; etc. Data for European countries were taken from the EU-SILC database (no data was available for Australia and Canada on this indicator).
3. Pre-school investment (0-5 years old) was calculated as the annual percentage of Gross Domestic Product (GDP) for each country. Data for all countries were taken from the OECD database.

Independent variable of interest

For peer review only - http://bmjopen.bmj.com/site/about/guidelines.xhtml

Austerity assessment: An indicator based on the Cyclically Adjusted Primary Balance (CAPB) published by the International Monetary Fund (IMF) [21] was used to analyse and identify the extent of austerity policy responses to the crisis in each participating country during the period 2009 to 2013[22], in general the last year with major spending cuts. The CAPB represents the cyclical component of the overall fiscal balance, computed as the difference between cyclical revenues and cyclical expenditures. It represents the average annual change in the cyclically adjusted primary balance, an estimate of what the difference between taxes and non-interest spending would be if the economy were at full employment. A high score equates to a higher level of austerity. CAPB was divided into tertiles representing high, medium and low level of austerity.

Co-variable:

Time period as a yearly dummy variable (2005 to the latest available data), and also stratified in 3 time periods - 2005-7 (pre-crisis); 2008-10 (economic crisis); 2012-15 (austerity and welfare period).

Data analysis

The analysis progressed in three stages. First we undertook a descriptive analysis of longitudinal trends in perinatal outcomes and the SDCH .

Secondly, we assessed the relationship between austerity category, and change in perinatal outcomes and SDCH, stratified in the 3 time periods.

Finally we used a longitudinal Generalised Estimating Equations (GEE) model, with model-based on robust standard errors[23], to allow for analysis of our correlated repeated outcome measurements (SDCH and perinatal outcomes). The independent variables included in the models were time-period, and CAPB. Time-period was included in the model as a yearly dummy variable (2005 to the latest available data), and also stratified in 3 time periods. Interaction terms were also included in the model to assess the influence of the time-period and the level of austerity. The analysis was conducted using STATA version 12.0.

All procedures were carried out following the data protection requirements of the European Parliament (Directive 95/46/EC of the European Parliament and of the Council of 24 October 1995 on the protection of individuals with regard to the processing of personal data and on the free movement of such data).

For peer review only

For peer review only - http://bmjopen.bmj.com/site/about/guidelines.xhtml

Results

Higher rates of LBW were seen in Greece, Spain and Portugal, while Iceland showed the lowest rates during the period 2005-15. Child poverty increased more for Greece and Spain than for the rest of countries, the latter especially showing an increasing trend at the end of the period. By contrast, Finland had lower rates of child poverty with a diminishing trend during the last years. Greece showed an increasing percentage of material deprivation in children from families with primary education level from the year 2009 onward, while Sweden showed the opposite trend (data not shown, available on request).

Table 1 shows the CAPB 2013-09 for countries included in the study, and stratified by level of austerity in tertiles. CAPB scores ranged from -0.43 (Sweden) to 3.43 (Greece).

Tables 2 and 3 show perinatal outcomes and SDCH indicators by country, and stratified according to the level of austerity, in 3 time periods. The high austerity group presents a trend to increasing LBW rates across the study periods, while the other groups show a slight decrease in 2012-15. IM shows a continuously diminishing trend in all 3 groups over the study period. Low austerity group of countries showed lower rates of child poverty and material deprivation than the intermediate and high austerity group and the rates remained relatively stable across the 3 study periods (i.e. percentage of material deprivation in the low austerity group was 14.4%; 14.6%; and 16%; while in the high austerity group was 15%; 15.8% and 24.9%, respectively). Pre-school investment increased in the low austerity group from 0.93% of the GDP to 1.16% in the last period (2012-15), while in the intermediate and high-austerity groups the increase was lower. Although Iceland is classified by the CAPB as a high austerity country, it is an outlier as its perinatal and SDCH rates are similar to other Nordic countries in the low austerity group.

Tables 4 and 5 show GEE models for perinatal and the SDCH outcomes analysed in 3 time-periods. CAPB was associated with LBW and there was a significant interaction in the period 2012-15, indicating that LBW increased more in the high austerity countries (interaction CAPB-period 2012-15, B: 5.27; p<0.001). No relationship was found between CAPB and IM. Material deprivation increased during the period 2012-15, and mainly in those countries with higher CAPB (interaction CAPB-period 2012-15, B: 5.62: p< 0.001). No association was found between pre-school investment and CAPB.

For peer review only - http://bmjopen.bmj.com/site/about/guidelines.xhtml

Discussion

To our knowledge, this is the first study exploring the impact of austerity imposed by governments in high income countries on perinatal outcomes and SDCH above and beyond the impact of the economic crisis itself. The study provides some evidence to suggest that austerity measures themselves have had an impact on children's health, independent of the economic crisis that led to austerity. The results support the hypothesis that those countries that have applied and maintained higher levels of austerity have experienced adverse consequences for children that will likely have implications for the future adult health of a generation.

The results of the present study corroborate other studies at the European general population level.[24] According to the latter, although there are many differences between countries, the analysis suggests that the interaction of fiscal austerity with economic shocks and weak social protection ultimately seems to produce greater social crisis. In general, the initial response to the crisis was increasing public spending during the years immediately following the crisis. Afterwards, the Greek, Spanish, and the UK governments modified public policy to reduce deficits by cutting public expenditure, particularly on welfare benefits, with adverse effects on SDCH. On the other hand, in other countries such as Iceland, besides its specific characteristics, governmental responses have attempted to gave prominence to redistribution, and try to protect middle-income and low-income groups.[19] This fact would explain, in part, the differences observed between the austerity group to which Iceland belongs and SDCH indicators.

A systematic review of the impact of the crisis on population health included a few studies on child health,[13] one of which found increased odds of LBW deliveries during the crisis in Iceland.[25] The results of our study are consistent with this finding particularly in the countries that applied the most severe austerity measures. The studies included in the systematic review by Parmar and colleagues are likely to have been too early to detect the full extent of the impact of the crisis and austerity measures on child health. Nevertheless, an increase in child mental health admissions and a high prevalence of mental health problems in children were described, and also poor well-being and an increase in the prevalence of overweight and obesity in poorest areas of the UK and among vulnerable children in Spain.[26-29]

This is one of the first studies that allowing the analysis of trends in SDCH and perinatal indicators starting before the economic crisis. Although it is an observational study with ecological data from each country analysed, the findings provide credible evidence on the impact of austerity in childhood. The main strengths of our study include the use of nationally representative data with standardised and comparable definitions and methods, and a study design that allowed us to analyse time trends starting in 2005, 3 years before the economic crisis, through to 2015.

Some limitations of the study deserve comment. First of all, countries included in the study may not allow the results to be generalised. Nevertheless, European countries from the Euro and non Euro areas were included, and also Australia and Canada as other OECD rich countries outside Europe. Future studies should extend the number of countries included to assess the influence of austerity in the remaining rich as well as middle and low income countries.

For peer review only - http://bmjopen.bmj.com/site/about/guidelines.xhtml

Secondly, those variables used to assess austerity, SDCH, and perinatal outcomes may have limited ability to discriminate specific measures taken by Governments. The austerity measure, the CAPB, used in this study, although being a generally accepted economic measure of austerity, may not adequately reflect national policies which institute general economic stringency at the same time as attempting to protect the most vulnerable groups, such as children. This might partly explain why Iceland, although having a CAPB suggesting a high level of austerity, shows trends more consistent with those for countries with intermediate and low levels of austerity. Another limitation of this data is the absence of variables that allow inequalities in the SDCH and perinatal outcomes within countries to be analysed. It is well known that indicators analysed as averages often mask differences between population groups. For example, IM showed trends to improve throughout the study period, but it has been shown to have increased in the UK in recent years in the most disadvantaged social classes.[30] Thirdly, the difficulties establishing causal associations between exposure and outcomes is well known especially in the case of international comparisons over time.

In summary, to our knowledge, this is the first study to attempt to distinguish the negative impact on perinatal outcomes and SDCH of austerity measures imposed by government in response to the economic crisis from the effects of the economic crisis itself. Our findings suggest that those governments that applied high levels of austerity have exacerbated the effects of 2008 economic crisis specifically increasing child poverty, material deprivation in families at most need, and perinatal outcomes such as LBW. The findings suggest the need to urgently protect vulnerable groups of children from the impact of austerity.

For peer review only - http://bmjopen.bmj.com/site/about/guidelines.xhtml

Contributors: LR and NS were involved in the initial conception and design of the study. LR extracted data and NS, DTR, GG and AH participated in the data analysis strategy. All authors were involved in the interpretation and discussion of results through call conferences. LR developed the first draft of the manuscript and all authors critically revised it and approved the final version. LR is study guarantor.

Funding: DTR is funded by the MRC on a Clinician Scientist Fellowship (MR/P008577/1). The study was not externally financed.

Competing interests: All authors have completed the ICMJE uniform disclosure form at www.icmje.org/coi_disclosure.pdf (available on request from the corresponding author), and have read and understood the BMJ policy on declaration of interests and declare the following interests: none.

Exclusive licence: The Corresponding Author has the right to grant on behalf of all authors and does grant on behalf of all authors, a worldwide licence to the Publishers and its licensees in perpetuity, in all forms, formats and media (whether known now or created in the future), to i) publish, reproduce, distribute, display and store the Contribution, ii) translate the Contribution into other languages, create adaptations, reprints, include within collections and create summaries, extracts and/or, abstracts of the Contribution, iii) create any other derivative work(s) based on the Contribution, iv) to exploit all subsidiary rights in the Contribution, v) the inclusion of electronic links from the Contribution to third party material where-ever it may be located; and, vi) licence any third party to do any or all of the above.

For peer review only

Table 1. Scores of the Cyclically Adjusted Primary Balance (CAPB), and stratified according to the level of austerity.

Country	CAPB	Level of austerity
Canada	.12	Low
Denmark	.02	Low
Finland	-0.1	Low
Germany	.08	Low
Norway	.14	Low
Sweden	-0.43	Low
Australia	.55	Intermediate
Austria	.58	Intermediate
Belgium	.35	Intermediate
France	.52	Intermediate
Italy	.64	Intermediate
Netherlands	.74	Intermediate
Greece	3.43	High
Iceland	1.75	High
Ireland	1.83	High
Portugal	1.68	High
Spain	1.93	High
United Kingdom	.86	High

CAPB: Cyclically adjusted primary balance.

For peer review only - http://bmjopen.bmj.com/site/about/guidelines.xhtml

Table 2 Low birth weight (LBW) and infant mortality (IM) in 3 time-periods (2005-07/ 2008-10 /2012-15), by country and according to the level of austerity in tertiles (low, intermediate and high).

**Level of austerity**	**Perinatal outcomes**					
	**LBW** (%)			**IM** (‰)		
	2005-07	2008-10	2012-15	2005-07	2008-10	2012-15
**Low**						
Canada	6.03	6.10	6.20	5.2	5	4.8
Denmark	5.30	5.30	5.25	4.0	3.5	3.65
Finland	4.23	4.23	4.15	2.8	2.5	2.02
Germany	6.83	6.86	6.75	3.9	3.46	3.27
Norway	4.98	5.20	4.62	3.1	2.86	2.4
Sweden	4.20	4.26	4.35	2.6	2.5	2.5
Total	***5.26***	***5.32***	***5.22***	***3.59***	***3.03***	***3.1***
**Intermediate**						
Australia	6.33	6.16	6.33	4.6	4.13	3.37
Austria	7.03	7.06	6.67	3.8	3.8	3.1
Belgium	6.96	6.93	6.90	3.9	3.63	3.5
France	6.65	6.73	6.33	3.8	3.76	3.57
Italy	6.76	7.10	7.35	3.2	3.1	2.87
Netherlands	6.76	5.83	5.80	4.5	3.8	3.8
Total	***6.74***	***6.63***	***6.56***	***3.95***	***3.7***	***3.36***
**High**						
Greece	8.86	9.33	9.32	3.7	3.2	3.6
Iceland	3.90	3.83	4.10	1.9	2.16	1.8
Ireland	4.93	5.00	5.55	3.6	3.43	3.42
Portugal	7.63	8.06	8.70	3.4	3.13	3.02
Spain	7.33	7.70	7.80	3.5	3.23	2.85
United Kingdom	7.36	7.03	6.95	4.9	4.43	3.92
Total	***6.66***	***6.82***	***7.07***	***3.5***	***3.26***	***3.1***

For peer review only - http://bmjopen.bmj.com/site/about/guidelines.xhtml

Table 3 Social determinants of child health (SDCH) in 3 time-periods (2005-07/ 2008-10 /2012-15), by country and according to the level of austerity in tertiles (low, intermediate and high).

Level of austerity	SDCH								
	Child poverty (%)			Material deprivation (%)			Pre-school investment (% of GDP)		
	2005-07	2008-10	2012-15	2005-07	2008-10	2012-15	2005-07	2008-10	2012-15
**Low**									
Canada	15.98	15.87	12.2	-	-	-	-	-	-
Denmark	8.33	8.8	8.3	14.2	7.5	13.8	1.29	1.26	1.37
Finland	8.96	10.5	8.87	12.8	14.1	11.87	0.88	0.99	1.10
Germany	11.5	13.56	13.4	24.8	30.0	30.52	0.37	0.42	0.55
Norway	8.63	8.9	8.37	8.6	11.5	14	0.83	1.12	1.22
Sweden	10.36	11.23	13.5	11.7	10.2	10	1.28	1.47	1.60
Total	***10.62***	***11.47***	***10.67***	***14.42***	***14.65***	***16.03***	***0.93***	***1.05***	***1.16***
**Intermediate**									
Australia	-	-	-	-	-	-	0.37	0.49	0.59
Austria	12.6	14.9	14.95	16.4	18.6	21.35	0.28	0.38	0.48
Belgium	14.6	14.73	15.72	22.3	21.0	24.35	0.60	0.64	0.75
France	13.4	14.83	16.37	16.5	22.5	23.7	1.10	1.14	1.25
Italy	22.63	22.13	23.77	15.7	17.9	29.12	0.52	0.54	0.53
Netherlands	11.7	11.13	11.4	10.3	9.1	9.27	0.54	0.82	0.71
Total	***14.9***	***15.5***	***16.4***	***16.23***	***17.81***	***21.55***	***0.56***	***0.66***	***0.71***
**High**									
Greece	21.96	22.43	27.02	20.8	27.3	50.82	-	-	-
Iceland	9.5	9.13	8.8	7.7	2.6	7.7	1.30	1.57	1.81
Ireland	18.33	16.26	17.12	17.5	19.1	26.12	0.28	0.43	0.50
Portugal	18.6	19.8	21.97	12.9	14.3	18.6	0.35	0.37	0.37
Spain	22.2	24.46	26.67	9.2	11.2	17.52	0.43	0.52	0.53
United Kingdom	19.96	18.93	18.1	22.4	20.8	28.82	0.79	0.77	0.77
Total	***18.4***	***18.5***	***19.9***	***15.09***	***15.8***	***24.93***	***0.63***	***0.73***	***0.79***

SDCH: social determinants of child health; GDP: Gross domestic product. Material deprivation in families with primary education level.

For peer review only - http://bmjopen.bmj.com/site/about/guidelines.xhtml

Table 4. Generalised estimating equation (GEE) of panel data of perinatal outcomes (low birth weight [LBW], and infant mortality [IM]) 2005-2015

	**Model 1 (crude)**		**Model 2 (adjusted by CAPB)**		**Model 3 Interactions (time-period*CAPB)**	
LBW						
Independent variables						
**Time period**						
2008-10	0.03	NS	0.03	NS	-0.05	NS
2012-15	0.05	NS	0.05	NS	-0.13	NS
**CAPB**			0.79	*0.006*	0.69	*0.02*
**Interaction terms**						
CAPB*2008-10					0.11	NS
CAPB*2012-15					0.24	*0.004*
Constant	6.22	*<0.001*	5.57	*<0.001*	5.27	*<0.001*
IM						
Independent variables						
**Time period**						
2008-10	-0.26	*<0.001*	-0.26	*<0.001*	-0.26	*0.002*
2012-15	-0.5	*<0.001*	-0.5	*<0.001*	-0.58	*<0.001*
**CAPB**			-0.05	NS	-0.08	NS
**Interaction terms**						
CAPB*2008-10					-0.002	NS
CAPB*2012-15					0.10	NS
Constant	3.68	*<0.001*	3.73	*<0.001*	3.76	*<0.001*

Reference category: years 2005-07. CAPB: Cyclically Adjusted Primary Balance; LBW: low birth weight; IM: infant mortality.

For peer review only - http://bmjopen.bmj.com/site/about/guidelines.xhtml

Table 5. Generalised estimating equation (GEE) of panel data. SDCH (child poverty 2005-2015, material deprivation 2005-2015, and pre-school investment 2005-2013)

	**Model 1 (crude)**		**Model 2 (adjusted by CAPB)**		**Model 3 Interactions (time-period*CAPB)**	
Child poverty						
	Coef.	p	Coef.	p	Coef.	p
Time period						
2008-10	0.47	NS	0.47	NS	0.67	NS
2012-15	0.95	NS	0.93	NS	0.18	NS
CAPB			3.62	*<0.001*	3.40	*<0.001*
**Interaction terms**						
CAPB*2008-10					-0.93	NS
CAPB*2012-15					0.91	0.07
Constant	14.21	*<0.001*	11.27	*<0.001*	11.46	*<0.001*
Material deprivation						
Independent variables						
**Time period**						
2008-10	0.86	NS	0.86	NS	0.01	NS
2012-15	5.86	*<0.001*	5.86	*<0.001*	0.92	NS
**CAPB**			2.37	NS	0.17	NS
**Interaction terms**						
CAPB*2008-10					0.96	NS
CAPB*2012-15					5.62	*<0.001*
Constant	15.23	*<0.001*	13.15	*<0.001*	15.08	*<0.001*
Pre-school investment						
Independent variables						
**Time period**						
2008-10	0.1	*<0.001*	0.1	*<0.001*	0.1	*0.006*
2012-13	0.18	*<0.001*	0.18	*<0.001*	0.19	*<0.001*
**CAPB**			-0.18	NS	-0.18	NS
**Interaction terms**						
CAPB*2008-10					0.008	NS
CAPB*2012-13					-0.01	NS
Constant	0.7	*<0.001*	0.83	*<0.001*	0.89	*<0.001*

Reference category: years 2005-07. CAPB: Cyclically Adjusted Primary Balance. SDCH: social determinants of child health.

For peer review only - http://bmjopen.bmj.com/site/about/guidelines.xhtml

References

1 Dávila-Quintana CD, González L-VB. Economic crisis and health. *Gac Sanit* 2009;23:261–5.

2 Bargain O, Callan T, Doorley K, Keane C. Changes in Income Distributions and the Role of Tax-Benefit Policy During the Great Recession: An International Perspective. *Fiscal Studies* 2017; 1–27.

3 Basu S, Carney M, Kenworthy NJ. Ten years after the financial crisis: The long reach of austerity and its global impacts on health. *Soc Sci Med* 2017; 1e5. http://dx.doi.org/10.1016/j.socscimed.2017.06.026

4 Ruckert A, Labont R. Health inequities in the age of austerity: The need for social protection policies. *Soc Sci Med* 2017;187:306-11. http://dx.doi.org/10.1016/j.socscimed.2017.03.029.

5 Flores M, García-Gómez P, Zunzunegui MV. Economic crisis, poverty and childhood. What are the expected short- and long-term effects for the "children of the crisis"? SESPAS report 2014. *Gac Sanit* 2014;28 Suppl 1:132-6. doi: 10.1016/j.gaceta.2014.02.021.

6 Barr B, Taylor-Robinson D. Recessions are harmful to health. *BMJ* 2016;354:i4631 doi: doi: 10.1136/bmj.i4631

7 Entwisle DR, Astone NM. Some Practical Guidelines for Measuring Youth's Race/Ethnicity and Socioeconomic Status. *Child Dev* 1993; 65:1521-40.

8 Dahlgren G, Whitehead M. Policies and strategies to promote social equity in health. Institute for future studies. (revised version) 2007:14.

9 Galobardes B, Lynch JW, Smith GD. Is the association between childhood socioeconomic circumstances and cause-specific mortality established? Update of a systematic review. *J Epidemiol Community Health* 2008;62:387–90.

10 Hirsch D, Spencer N. Unhealthy lives; intergenerational links between child poverty and poor health in the UK, summary briefing. End Child Poverty Now Policy Briefing; 2008. http://www.donaldhirsch.com/unhealthylives.pdf.

11 Non Al, Román JC, Gross CL, Gilman SE, Loucks EB, Buka SL, et al. Early childhood social disadvantage is associated with poor health behaviours in adulthood. *Ann Hum Biol* 2016; 43(2): 144–153. doi:10.3109/03014460.2015.1136357.

For peer review only - http://bmjopen.bmj.com/site/about/guidelines.xhtml

12 Rajmil L, Sanmamed MJF, Choonara I, Faresjö T, Hjern A, Kozyrskyj AL, on Behalf of the International Network for Research in Inequalities in Child Health (INRICH). Impact of the 2008 Economic and Financial Crisis on Child Health: A Systematic Review. *Int J Env Res Public Health* 2014;11(6):6528–46.

13 Parmar D, Stavropoulou C, Ioannidis JPA. Health outcomes during the financial crisis in Europe: systematic literature review. *BMJ* 2016;354:i4588.

14 Taylor-Robinson D, Whitehead M, Barr B. Great leap backwards. *BMJ* 2014;349:g7350.

15 Rajmil L, Artazcoz L, García-Gómez P, Flores M, Hernández-Aguado I. Spanish children are most affected by the economic crisis, says the Spanish Society of Public Health (SESPAS). *BMJ* 2015;350:h1945.

16 Unicef Office of Research. Children of the recession: the impact of the economic crisis, on child well-being in rich countries; 2014. www.unicef-irc.org/publications/pdf/rc12-engweb.pdf.

17 Sdona E, Papamichail D, Ragkou E, Briana DD, Malamitsi-Puchner A, Panagiotopoulos T. Greek economic crisis and impaired perinatal parameters: experience from a public maternity hospital. *J Matern Fetal Neonatal Med* 2017;4:1-5. doi: 10.1080/14767058.2017.1342803.

18 Varea C, Terán LM, Bernis C, Bogin B, González-González A. Is the economic crisis affecting birth outcome in Spain? Evaluation of temporal trend in underweight at birth (2003–2012). *Ann Hum Biol* 2016;43(2):169–82 DOI: 10.3109/03014460.2015.1131847

19 Gunnlaugsson G. Child health in Iceland before and after the economic collapse in 2008. *Arch Dis Child* 2015;0:1–8. doi:10.1136/archdischild-2014-307196.

20 Cantillon B, Chzhen Y, Handa S, Nolan B. Children of Austerity. Impact of the Great Recession on Child Poverty in Rich Countries. UNICEF: Oxford University Press; 2017.

21 International Monetary Fund (IMF). Fiscal Monitor. Achieving More with Less. IMF: 2017. http://www.imf.org/en/Publications/FM/Issues/2017/04/06/fiscal-monitor-april-2017.

22 Krugman P. The case for cuts was a lie. Why does Britain still believe it? The austerity delusion. The Guardian: 29th April 2015. https://www.theguardian.com/business/ng-interactive/2015/apr/29/the-austerity-delusion

For peer review only - http://bmjopen.bmj.com/site/about/guidelines.xhtml

23 Ballinger G. Using generalized estimation equations for longitudinal data analysis. *Org Res Methods* 2004;7:127–50. doi:10.1177/1094428104263672.

24 Karanikolos M, Mladovsky P, Cylus J, Thomson S, Basu S, Stuckler D, et al. Financial crisis, austerity, and health in Europe. *Lancet* 2013;381:1323–31.

25 Eiríksdóttir VH, Ásgeirsdóttir TL, Bjarnadóttir RI, Kaestner R, Cnattingius S, Valdimarsdóttir UA. Low birth weight, small for gestational age and preterm births before and after the economic collapse in Iceland: a population based cohort study. *PLoS One* 2013;8:e80499. doi:10.1371/journal.pone.0080499.

26 Novoa AM, Ward J, Malmusi D, Díaz F, Darnell M, Trilla C, et al. Condicions de vida, habitatge i salut. Mostra de persones ateses per Càritas Diocesana de Barcelona. Barcelona; Càritas Diocesana de Barcelona; 2013.

27 Plataforma de afectados por la Hipoteca. Te quedarás en la Oscuridad. Desahucio, familia e infancia desde un enfoque de derechos. Madrid: PAH; 2016. http://pahmadrid.org/infancia/estudio-te-quedaras-en-la-oscuridad.

28 Rajmil L, Medina-Bustos A, Fernández de Sanmamed MJ, Mompart-Penina A. Impact of the economic crisis on children’s health in Catalonia: a before–after approach. *BMJ* Open 2013; 3:e003286. doi:10.1136/bmjopen-2013-003286.

29 McKenna C, Law C, Pearce A. Increased household financial strain, the Great Recession and child health—findings from the UK Millennium Cohort Study. *BMJ Open* 2017;7:e015559. doi:10.1136/bmjopen-2016-015559

30 Taylor-Robinson D, Barr B. Death rate now rising in UK’s poorest infants. *BMJ* 2017;357:j2258 doi: 10.1136/bmj.j2258.

For peer review only

For peer review only - http://bmjopen.bmj.com/site/about/guidelines.xhtml

BMJ Open

Trends in social determinants of child health and perinatal outcomes in European countries 2005-2015 by level of austerity imposed by governments: a repeat cross-sectional analysis of routinely available data

Journal:	*BMJ Open*
Manuscript ID	bmjopen-2018-022932.R1
Article Type:	Research
Date Submitted by the Author:	19-Jul-2018
Complete List of Authors:	Rajmil, Luis; Retired,<br>Taylor-Robinson, David; University of Liverpool,<br>Gunnlaugsson, Geir; Faculty of Social and Human Sciences University of Iceland<br>Hjern, Anders; Centre for Health Equity Studies (CHESS) Stockholm University/Karolinska Institutet<br>Spencer, Nick; University of Warwick, School of Health and Social Studies
**Primary Subject Heading**:	Health policy
Secondary Subject Heading:	Public health
Keywords:	austerity, child health, perinatal outcomes, social determinants of health

SCHOLARONE™ Manuscripts

For peer review only

Trends in social determinants of child health and perinatal outcomes in European countries 2005-2015 by level of austerity imposed by governments: a repeat cross-sectional analysis of routinely available data

Authors:
Luis Rajmil,[1] David Taylor-Robinson,[2] Geir Gunnlaugsson,[3] Anders Hjern,[4] Nick Spencer[5]

[1] Retired, paediatrician and public health and epidemiology specialist. Homer 22 1rst 1 Barcelona Spain
[2] Clinical Senior Lecturer, Department of Public Health and Policy, Whelan Building University of Liverpool ,Liverpool, UK
[3] Faculty of Social and Human Sciences, University of Iceland, Reykjavik, Iceland
[4] Centre for Health Equity Studies (CHESS), Stockholm University/Karolinska Institutet, SE-106 91, Stockholm, Sweden
[5] Emeritus Professor of Child Health, Division of Mental Health and Wellbeing, Warwick Medical School University of Warwick, Coventry, CV4 9JD, UK

Corresponding author:
Luis Rajmil Homer 22, 1rst, 1. Barcelona 08023. Spain
E-mail: 12455lrr@comb.cat
Phone +34 93 4173243

For peer review only - http://bmjopen.bmj.com/site/about/guidelines.xhtml

Abstract

Objective To assess whether the level of austerity implemented by national governments was associated with adverse trends in perinatal outcomes and the social determinants of children's health (SDCH) in rich countries

Design Longitudinal ecological study of country level time trends in perinatal outcomes and SDCH and from 2005-2015

Setting and participants 16 European countries using available data from the International Monetary Fund (IMF), the Organisation for Economic Co-operation and Development (OECD), and Eurostat.

Main outcome measures Trends in perinatal outcomes (low birth weight (LBW); infant mortality (IM)) and the social determinants of child health (SDCH: child poverty rates; severe material deprivation in families with primary education; pre-school investment) in 3 time-periods: 2005-07, 2008-10 and 2012-15. Outcomes were compared according to the cyclically adjusted primary balance (CAPB, differences between 2013 and 2009) as a measure of austerity, stratified in tertiles. Generalised estimating equation (GEE) models of repeated measures were used to assess time-trend differences in 3 periods.

Results Countries with higher levels of austerity had worse outcomes, mainly at the last study period. Material deprivation increased during the period 2012-15 in those countries with higher CAPB (interaction CAPB-period 2012-15, B: 5.62: p< 0.001), as did LBW (interaction CAPB-period 2012-15, B: 0.25; p0.004).

Conclusions Countries that implemented more severe austerity measures have experienced increasing LBW and, for families with primary education also increasing material deprivation, worsening the negative impact of economic crisis. Reversing austerity policies that impact children is likely to improve child health outcomes.

Words in the text: 2750
Words in the abstract: 243
References: 33
Tables: 5

For peer review only

For peer review only - http://bmjopen.bmj.com/site/about/guidelines.xhtml

Strengths and limitations of this study

- This is one of the first studies to analyse trends in SDCH and perinatal indicators starting before the economic crisis.
- The main strengths of the study include the use of nationally representative data with standardised and comparable definitions and methods, and a study design that allowed us to analyse time trends starting in 2005, 3 years before the economic crisis, through to 2015.
- Countries included in the study may not allow the results to be generalised
- Variables used to assess austerity, SDCH, and perinatal outcomes may have limited ability to discriminate specific measures taken by Governments

For peer review only

For peer review only - http://bmjopen.bmj.com/site/about/guidelines.xhtml

Introduction

The global financial crisis starting in 2008 has had a great economic impact around the world, although the impact in each country depends on several factors, such as the starting point, mechanisms of social protection and social transfers, and the measures adopted by governments to deal with the crisis.[1] During the period 2008-10 the initial financial crisis was followed by a downturn in economic activity, resulting in evictions, foreclosures, and prolonged unemployment in many countries. From 2010-11 onward the pressure to adopt and enforce austerity measures, mainly within the European Union and worldwide, has been levied by global financial institutions such as the International Monetary Fund.[2-4] These austerity measures taken by governments were neither homogeneous nor similarly implemented in rich countries; some protected public sector programs and systems while others instituted large budget cuts in education, health and other public services.

Assessing the health effects of the crisis is challenging given the difficulty establishing association of exposure and outcomes. There is a lag time between exposure to the effects of a severe economic downturn and adverse health outcomes[5] and, in the case of exposure in early childhood, the adverse effects may not emerge until adulthood.[6] Childhood is an especially vulnerable period to the main determinants of health, such as living conditions, family income, employment, education, housing, access to health services, among other social determinants of child health (SDCH) to individual life style factors. All factors related to financial capital (i.e. family income), human capital (i.e. education), and social capital (family working conditions) have potential influence on future child health and development.[7, 8] Moreover, there is overwhelming evidence for the profound effects of social factors and SDCH on health throughout childhood and into adulthood.[9-11]

Findings from studies to date on the impact of the economic crisis on SDCH and perinatal outcomes, carried out at national and international level show foetal and childhood populations as population groups most affected by the crisis.[12-16] Vulnerability starts in the prenatal period with adverse effects on perinatal outcomes.[17-19] Most studies of the impact of economic crisis on health have not distinguished between economic crises themselves and policy responses to these crises.[6] Furthermore there is a paucity of literature on the impact of austerity measures imposed by government in response to the economic crisis on perinatal outcomes and SDCH.[20] The analysis of time trends beginning before the crisis until the post-crisis period allows evaluation of political responses and their impact on SDCH and perinatal health.

The aim of the study was therefore to assess time trends in perinatal outcomes and SDCH by level of austerity enforced by governments. We hypothesise that those countries that implemented and maintained high levels of austerity would show statistically significant differences in adverse trends in perinatal outcomes and SDCH compared with countries imposing lower levels of austerity.

For peer review only - http://bmjopen.bmj.com/site/about/guidelines.xhtml

Methods

We undertook a longitudinal ecological study of trends in perinatal outcomes and SDCH at country level, and assessed how these trends varied according to the level of austerity measures implemented. Sixteen European countries from the European Economic Area (EEA) were included in the study, on the basis of routinely available data for the period 2005-2015 pertaining to perinatal outcomes and SDCH. Luxembourg was excluded due to its high level of economic development, which not necessarily reflects the real wealth of residents. Post-communist countries were also excluded. The included countries are evenly distributed across low, intermediate and high austerity measures.

Outcome variables

Perinatal outcomes: Data on perinatal outcomes were taken from the Organisation for Economic Co-operation and Development (OECD) Family Database (http://www.oecd.org/els/family/database.htm).

1. Low birth weight (LBW) was defined as the number of live births weighing less than 2500 grs divided by the total number of live births.
2. Infant mortality: deaths of children aged less than one year per 1000 live births (no minimum threshold of gestation period or birth weight).

Social determinants of child health (SDCH):

1. Child poverty was defined as the percentage of children living in households with income below 60% of the median. Data were taken from the European Union-Survey of Income and Living conditions (EU-SILC) (http://ec.europa.eu/eurostat/web/microdata/european-union-statistics-on-income-and-living-conditions).
2. Severe material deprivation rate was defined as the proportion of children under 18 years of age living in families with primary level of education which cannot afford to pay for at least four out of the nine items considered basic, such as having arrears on mortgage or rent payments, utility bills, hire purchase instalments or other loan payments; not being able to afford one week's annual holiday away from home; not being able to afford a meal with meat, chicken, fish (or vegetarian equivalent) every second day; etc. Data were taken from the EU-SILC database.
3. Pre-school investment (0-5 years old) was calculated as the annual percentage of Gross Domestic Product (GDP) for each country. Data were taken from the OECD database.

Independent variable of interest

For peer review only - http://bmjopen.bmj.com/site/about/guidelines.xhtml

Austerity assessment: An indicator based on the Cyclically Adjusted Primary Balance (CAPB) published by the International Monetary Fund (IMF) [21] was used to analyse and identify the extent of austerity policy responses to the crisis in each participating country during the period 2009 to 2013[22], in general the last year with major spending cuts. The CAPB represents the cyclical component of the overall fiscal balance, computed as the difference between cyclical revenues and cyclical expenditures. It represents the average annual change in the cyclically adjusted primary balance, an estimate of what the difference between taxes and non-interest spending would be if the economy were at full employment. A high score equates to a higher level of austerity. CAPB was divided into tertiles representing high, medium and low level of austerity.

Co-variable:

Time period (2005 to the latest available data), was stratified in 3 time periods - 2005-7 (pre-crisis); 2008-10 (economic crisis); 2012-15 (austerity and welfare period or post-crisis period).

Data analysis

The analysis progressed in three stages. First we undertook a descriptive analysis of longitudinal trends in perinatal outcomes and the SDCH.

Secondly, we assessed the relationship between austerity category, and change in perinatal outcomes and SDCH, stratified in the 3 time periods.

Finally we used a longitudinal Generalised Estimating Equations (GEE) model, based on robust standard errors[23], to allow for analysis of our correlated repeated outcome measurements (SDCH and perinatal outcomes; see Figure 1 in the Supplementary material). GEE has been advocated as a tool for evaluating policy change and natural experiments.[24] The independent variables included in the models were time-period, and CAPB. Time-period was stratified in 3 time periods for our final model. Interaction terms were also included in the model to assess the influence of the time-period and the level of austerity. The analysis was conducted using STATA version 12.0.

All procedures were carried out following the data protection requirements of the European Parliament (Directive 95/46/EC of the European Parliament and of the Council of 24 October 1995 on the protection of individuals with regard to the processing of personal data and on the free movement of such data). Ethical approval was not required for this study as it used routinely available aggregated data.

Patient and Public Involvement

There was no patient or public involvement in the design of this study.

For peer review only - http://bmjopen.bmj.com/site/about/guidelines.xhtml

Results

Higher rates of LBW were seen in Greece, Spain and Portugal, while Iceland showed the lowest rates during the period 2005-15. Child poverty increased more for Greece and Spain than for the rest of countries, the latter especially showing an increasing trend at the end of the period. By contrast, Finland had lower rates of child poverty with a diminishing trend during the last years. Greece showed an increasing percentage of material deprivation in children from families with primary education level from the year 2009 onward, while Sweden showed the opposite trend (Figures2 to 6 in the Supplementary material).

Table 1 shows the CAPB 2013-09 for countries included in the study, and stratified by level of austerity in tertiles. CAPB scores ranged from -0.43 (Sweden) to 3.43 (Greece).

Perinatal and SDCH outcomes according to the level of austerity

Tables 2 and 3 show perinatal outcomes and SDCH indicators by country, and stratified according to the level of austerity, in 3 time periods. The high austerity group presents a trend to increasing LBW rates across the study periods, while the other groups show a slight decrease in 2012-15. IM shows a continuously diminishing trend in all 3 groups over the study period. Low austerity group of countries showed lower rates of child poverty and material deprivation than the intermediate and high austerity group and the rates remained relatively stable across the 3 study periods (i.e. percentage of material deprivation in the low austerity group was 14.42%; 14.65%; and 16.03%; while in the high austerity group was 15.09%; 15.80% and 24.93%, respectively). Pre-school investment increased in the low austerity group from 0.93% of the GDP to 1.16% in the last period (2012-15), while in the intermediate and high-austerity groups the increase was lower. Although Iceland is classified by the CAPB as a high austerity country, it is an outlier as its perinatal and SDCH rates are similar to other Nordic countries in the low austerity group.

Multivariate models

Tables 4 and 5 show GEE models for perinatal and SDCH outcomes analysed in 3 time-periods. CAPB was associated with LBW and there was a significant interaction in the period 2012-15, indicating that LBW increased more in the high austerity countries (interaction CAPB-period 2012-15, B: 0.25; p0.004). No relationship was found between CAPB and IM. Material deprivation increased during the period 2012-15, and mainly in those countries with higher CAPB (interaction CAPB-period 2012-15, B: 5.62: p< 0.001). No association was found between pre-school investment and CAPB.

For peer review only - http://bmjopen.bmj.com/site/about/guidelines.xhtml

Discussion

To our knowledge, this is the first study exploring the impact of austerity imposed by governments in high income countries on perinatal outcomes and SDCH above and beyond the impact of the economic crisis itself. The study provides some evidence to suggest that austerity measures themselves have had an impact on children's health, independent of the economic crisis that led to austerity. The results support the hypothesis that those countries that have applied and maintained higher levels of austerity have experienced adverse consequences for children that will likely have implications for the future adult health of a generation.

The results of the present study corroborate other studies at the European general population level. [25] According to the latter, although there are many differences between countries, the analysis suggests that the interaction of fiscal austerity with economic shocks and weak social protection ultimately seems to produce greater social crisis. In general, the initial response to the crisis was increasing public spending during the years immediately following the crisis, mainly in those countries with increasing needs in vulnerable population groups (i.e. high unemployment rates). Afterwards, the Greek, Spanish, and the UK governments modified public policy to reduce deficits by cutting public expenditure, particularly on welfare benefits, with adverse effects on SDCH. On the other hand, in other countries such as Iceland, besides its specific characteristics, governmental responses have attempted to gave prominence to redistribution, and try to protect middle-income and low-income groups.[19] This fact would explain, in part, the differences observed between the austerity group to which Iceland belongs and SDCH indicators.

A systematic review of the impact of the crisis on population health included a few studies on child health,[13] one of which found increased odds of LBW deliveries during the crisis in Iceland.[26] It is worth noting that other variables should be taken into account when analysing perinatal outcomes such as LBW. For example age of mother at birth, origin, social class, rates of multiple births, etc., although our data do not allow us to go further into these mechanisms affecting maternal environment and birth outcomes, which should be the subject of future studies. In any case, the results of our study are consistent with the finding of the mentioned study, particularly in the countries that applied the most severe austerity measures. The studies included in the systematic review by Parmar and colleagues are likely to have been too early to detect the full extent of the impact of the crisis and austerity measures on child health. Nevertheless, an increase in child mental health admissions and a high prevalence of mental health problems in children were described, and also poor well-being and an increase in the prevalence of overweight and obesity in poorest areas of the UK and among vulnerable children in Spain.[27-30]

A recent study showed that the rate of increase in life expectancy in 25 European countries slowed in 2011-2015 regarding 2006-2010.[31] The relationship between high austerity and LBW but not IM in the present study could be associated to lag effects, as suggested by a recent rise in IM in the UK over last 2 years,[32] and also the large numbers and much less power to detect changes in IM. These results reinforce the need for long-term monitoring of perinatal indicators such as IM.

For peer review only - http://bmjopen.bmj.com/site/about/guidelines.xhtml

This is one of the first studies to analyse trends in SDCH and perinatal indicators starting before the economic crisis. Although it is an observational study with ecological data from each country analysed, the findings provide credible evidence on the impact of austerity in childhood. The main strengths of our study include the use of nationally representative data with standardised and comparable definitions and methods, and a study design that allowed us to analyse time trends starting in 2005, 3 years before the economic crisis, through to 2015. Although the starting period, the extent and implementation of austerity measures might differ between countries and periods, the results are plausible and show a specific impact on SDCH and perinatal outcomes.

Some limitations of the study deserve comment. First of all, countries included in the study may not allow the results to be generalised. Nevertheless, European countries from the Euro and non Euro areas were included. Future studies should extend the number of countries included to assess the influence of austerity in the remaining rich as well as middle and low income countries. Second, those variables used to assess austerity, SDCH, and perinatal outcomes may have limited ability to discriminate specific measures taken by Governments. Moreover, validity and reliability of SDCH and perinatal indicators would be affected by country variability on data collection, etc. Nevertheless, both Eurostat and OECD make effort to continuously improve the quality of data collection (see http://ec.europa.eu/eurostat/en/web/products-statistical-working-papers/-/KS-RA-12-018 and https://data.oecd.org/healthstat/infant-mortality-rates.htm as examples). The austerity measure, the CAPB, used in this study to differentiate policy responses, although being a generally accepted economic measure of austerity, may not adequately reflect national policies which institute general economic stringency at the same time as attempting to protect the most vulnerable groups, such as children. This might explain why Iceland, although having a CAPB suggesting a high level of austerity, shows trends more consistent with those for countries with intermediate and low levels of austerity. Another limitation of this data is the absence of variables that allow inequalities in the SDCH and perinatal outcomes within countries to be analysed. It is well known that indicators analysed as averages often mask differences between population groups. Moreover, aggregate data do not allow adjusting LBW or IM by relevant variables such as parity, multiplicity or type of birth. In this sense, the sustained rise in multiple pregnancies associated with an increased access to assisted reproductive technology, a general deterioration in neonatal indicators such as LBW, and a decrease in fertility since recession with changing socioeconomic status patterning, may underestimate the recession effect. For example, as previously mentioned, IM showed decreasing trends throughout the study period, but has been shown to have increased in the UK in recent years in the most disadvantaged social classes.[33] Third, the difficulties establishing causal associations between exposure and outcomes is well known especially in the case of international comparisons over time. Finally, as a result of the study design, the ecological fallacy cannot be ruled out.

In summary, to our knowledge, this is the first study to attempt to distinguish the negative impact on perinatal outcomes and SDCH of austerity measures imposed by government in response to the economic crisis from the effects of the economic crisis itself.

For peer review only - http://bmjopen.bmj.com/site/about/guidelines.xhtml

In conclusion, our findings suggest that those governments that applied high levels of austerity have exacerbated the effects of 2008 economic crisis on children specifically increasing child poverty, material deprivation in families at most need, and perinatal outcomes such as LBW. The findings suggest the need to urgently protect vulnerable groups of children from the impact of austerity.

For peer review only

For peer review only - http://bmjopen.bmj.com/site/about/guidelines.xhtml

Contributors: LR and NS were involved in the initial conception and design of the study. LR extracted data and NS, DTR, GG and AH participated in the data analysis strategy. All authors were involved in the interpretation and discussion of results through call conferences. LR developed the first draft of the manuscript and all authors critically revised it and approved the final version. LR is study guarantor.

Funding: DTR is funded by the MRC on a Clinician Scientist Fellowship (MR/P008577/1). The study was not externally financed.

Competing interests: All authors have completed the ICMJE uniform disclosure form at www.icmje.org/coi_disclosure.pdf (available on request from the corresponding author), and have read and understood the BMJ policy on declaration of interests and declare the following interests: none.

Exclusive licence: The Corresponding Author has the right to grant on behalf of all authors and does grant on behalf of all authors, a worldwide licence to the Publishers and its licensees in perpetuity, in all forms, formats and media (whether known now or created in the future), to i) publish, reproduce, distribute, display and store the Contribution, ii) translate the Contribution into other languages, create adaptations, reprints, include within collections and create summaries, extracts and/or, abstracts of the Contribution, iii) create any other derivative work(s) based on the Contribution, iv) to exploit all subsidiary rights in the Contribution, v) the inclusion of electronic links from the Contribution to third party material where-ever it may be located; and, vi) licence any third party to do any or all of the above.

Data sharing statement: The data is available from the corresponding author on request

For peer review only

Table 1. Scores of the Cyclically Adjusted Primary Balance (CAPB), and stratified according to the level of austerity.

Country	CAPB	Level of austerity
Denmark	.02	Low
Finland	-0.1	Low
Germany	.08	Low
Norway	.14	Low
Sweden	-0.43	Low
Austria	.58	Intermediate
Belgium	.35	Intermediate
France	.52	Intermediate
Italy	.64	Intermediate
Netherlands	.74	Intermediate
Greece	3.43	High
Iceland	1.75	High
Ireland	1.83	High
Portugal	1.68	High
Spain	1.93	High
United Kingdom	.86	High

CAPB: Cyclically adjusted primary balance: the average annual change in the difference between taxes and non-interest spending 2013- 2009 would be if the economy were at full employment. Higher score corresponds to higher level of austerity.

For peer review only

Table 2 Low birth weight (LBW) and infant mortality (IM) in 3 time-periods (2005-07/ 2008-10 /2012-15), by country and according to the level of austerity in tertiles (low, intermediate and high).

**Level of austerity**	**Perinatal outcomes**					
	**LBW** (%)			**IM** (‰)		
	2005-07	2008-10	2012-15	2005-07	2008-10	2012-15
**Low**						
Denmark	5.30	5.30	5.25	3.96	3.50	3.65
Finland	4.23	4.23	4.15	2.83	2.50	2.02
Germany	6.83	6.86	6.75	3.90	3.46	3.27
Norway	4.98	5.20	4.62	3.13	2.86	2.40
Sweden	4.20	4.26	4.35	2.56	2.50	2.50
Total	***5.10***	***5.17***	***5.02***	***3.28***	***2.96***	***2.76***
**Intermediate**						
Austria	7.03	7.06	6.67	3.83	3.80	3.10
Belgium	6.96	6.93	6.90	3.90	3.63	3.50
France	6.65	6.73	6.33	3.80	3.76	3.57
Italy	6.76	7.10	7.35	3.20	3.10	2.87
Netherlands	6.76	5.83	5.80	4.46	3.80	3.80
Total	***6.83***	***6.73***	***6.81***	***3.84***	***3.61***	***3.32***
**High**						
Greece	8.86	9.33	9.32	3.66	3.20	3.60
Iceland	3.90	3.83	4.10	1.90	2.16	1.80
Ireland	4.93	5.00	5.55	3.63	3.43	3.42
Portugal	7.63	8.06	8.70	3.40	3.13	3.02
Spain	7.33	7.70	7.80	3.53	3.23	2.85
United Kingdom	7.36	7.03	6.95	4.90	4.43	3.92
Total	***6.66***	***6.82***	***7.07***	***3.5***	***3.26***	***3.1***

For peer review only - http://bmjopen.bmj.com/site/about/guidelines.xhtml

Table 3 Social determinants of child health (SDCH) in 3 time-periods (2005-07/ 2008-10 /2012-15), by country and according to the level of austerity in tertiles (low, intermediate and high).

**Level of austerity**	SDCH								
	Child poverty (%)			Material deprivation (%)			Pre-school investment (% of GDP)		
	2005-07	2008-10	2012-15	2005-07	2008-10	2012-15	2005-07	2008-10	2012-15
**Low**									
Denmark	8.33	8.81	8.30	14.20	7.53	13.80	1.29	1.26	1.37
Finland	8.96	10.50	8.87	12.80	14.06	11.87	0.88	0.99	1.10
Germany	11.50	13.56	13.40	24.83	30.00	30.52	0.37	0.42	0.55
Norway	8.63	8.90	8.37	8.56	11.50	14.00	0.83	1.12	1.22
Sweden	10.36	11.23	13.5	11.73	10.20	10.00	1.28	1.47	1.60
Total	***9.55***	***10.59***	***10.48***	***14.42***	***14.65***	***16.03***	***0.93***	***1.05***	***1.16***
**Intermediate**									
Austria	12.63	14.90	14.95	16.49	18.63	21.35	0.28	0.38	0.48
Belgium	14.60	14.73	15.72	22.36	21.0	24.35	0.60	0.64	0.75
France	13.40	14.83	16.37	16.53	22.53	23.70	1.10	1.14	1.25
Italy	22.63	22.13	23.77	15.70	17.86	29.12	0.52	0.54	0.53
Netherlands	11.70	11.32	11.40	10.30	9.06	9.27	0.54	0.82	0.71
Total	***14.9***	***15.5***	***16.4***	***16.23***	***17.81***	***21.55***	***0.60***	***0.70***	***0.74***
**High**									
Greece	21.96	22.43	27.02	20.83	27.33	50.82	-	-	-
Iceland	9.50	9.13	8.80	7.76	2.60	7.70	1.30	1.57	1.81
Ireland	18.33	16.26	17.12	17.53	19.10	26.12	0.28	0.43	0.50
Portugal	18.60	19.80	21.97	12.93	14.33	18.60	0.35	0.37	0.37
Spain	22.60	24.46	26.67	9.23	11.16	17.52	0.43	0.52	0.53
United Kingdom	19.96	18.93	18.10	22.4	20.8	28.82	0.79	0.77	0.77
Total	***18.4***	***18.5***	***19.9***	***15.09***	***15.80***	***24.93***	***0.63***	***0.73***	***0.79***

SDCH: social determinants of child health; GDP: Gross domestic product. Material deprivation in families with primary education level.

For peer review only

Table 4. Generalised estimating equation (GEE) of panel data of perinatal outcomes (low birth weight [LBW], and infant mortality [IM]) 2005-2015

	Model 1 (crude)		Model 2 (adjusted by CAPB)		Model 3 Interactions (time-period*CAPB)	
LBW	**Coef.**	**p**	**Coef.**	**p**	**Coef.**	**p**
Independent variables						
**Time period**						
2008-10	0.04	NS	0.04	NS	-0.05	NS
2012-15	0.05	NS	0.05	NS	-0.16	NS
**CAPB**			0.8	*0.009*	0.7	*0.02*
**Interaction terms**						
CAPB*2008-10					0.11	NS
CAPB*2012-15					0.25	*0.004*
Constant	6.23	*<0.001*	5.51	*<0.001*	5.27	*<0.001*
IM						
Independent variables						
**Time period**						
2008-10	-0.256	*<0.001*	-0.25	*<0.001*	-0.25	*0.002*
2012-15	-0.46	*<0.001*	-0.32	*<0.001*	-0.55	*<0.001*
**CAPB**			-0.03	NS	-0.0028	NS
**Interaction terms**						
CAPB*2008-10					-0.002	NS
CAPB*2012-15					0.10	NS
Constant	3.53	*<0.001*	3.50	*<0.001*	3.53	*<0.001*

Reference category: years 2005-07. CAPB: Cyclically Adjusted Primary Balance; LBW: low birth weight; IM: infant mortality.

For peer review only

For peer review only - http://bmjopen.bmj.com/site/about/guidelines.xhtml

Table 5. Generalised estimating equation (GEE) of panel data. SDCH (child poverty 2005-2015, material deprivation 2005-2015, and pre-school investment 2005-2013)

	**Model 1 (crude)**		**Model 2 (adjusted by CAPB)**		**Model 3 Interactions (time-period*CAPB)**	
Child poverty						
	Coef.	p	Coef.	p	Coef.	p
Time period						
2008-10	0.51	NS	0.51	NS	0.75	NS
2012-15	1.29	0.01	1.29	<0.001	0.69	NS
CAPB			3.60	*<0.001*	3.46	*<0.001*
**Interaction terms**						
CAPB*2008-10					-0.27	NS
CAPB*2012-15					0.69	0.06
Constant	14.6	*<0.001*	11.44	*<0.001*	11.57	*<0.001*
Material deprivation						
Independent variables						
**Time period**						
2008-10	0.86	NS	0.86	NS	0.01	NS
2012-15	5.86	*<0.001*	5.86	*<0.001*	0.92	NS
**CAPB**			2.37	NS	0.17	NS
**Interaction terms**						
CAPB*2008-10					0.96	NS
CAPB*2012-15					5.62	*<0.001*
Constant	15.23	*<0.001*	13.15	*<0.001*	15.08	*<0.001*
Pre-school investment						
Independent variables						
**Time period**						
2008-10	0.1	*<0.001*	0.1	*<0.001*	0.1	*0.006*
2012-13	0.18	*<0.001*	0.18	*<0.001*	0.18	*<0.001*
**CAPB**			-0.19	NS	-0.19	NS
**Interaction terms**						
CAPB*2008-10					0.008	NS
CAPB*2012-13					-0.01	NS
Constant	0.72	*<0.001*	0.85	*<0.001*	0.85	*<0.001*

Reference category: years 2005-07. CAPB: Cyclically Adjusted Primary Balance. SDCH: social determinants of child health.

For peer review only - http://bmjopen.bmj.com/site/about/guidelines.xhtml

References

1 Dávila-Quintana CD, González L-VB. Economic crisis and health. *Gac Sanit* 2009;23:261–5.

2 Bargain O, Callan T, Doorley K, Keane C. Changes in Income Distributions and the Role of Tax-Benefit Policy During the Great Recession: An International Perspective. *Fiscal Studies* 2017; 1–27.

3 Basu S, Carney M, Kenworthy NJ. Ten years after the financial crisis: The long reach of austerity and its global impacts on health. *Soc Sci Med* 2017; 1e5. http://dx.doi.org/10.1016/j.socscimed.2017.06.026

4 Ruckert A, Labont R. Health inequities in the age of austerity: The need for social protection policies. *Soc Sci Med* 2017;187:306-11. http://dx.doi.org/10.1016/j.socscimed.2017.03.029.

5 Flores M, García-Gómez P, Zunzunegui MV. Economic crisis, poverty and childhood. What are the expected short- and long-term effects for the "children of the crisis"? SESPAS report 2014. *Gac Sanit* 2014;28 Suppl 1:132-6. doi: 10.1016/j.gaceta.2014.02.021.

6 Barr B, Taylor-Robinson D. Recessions are harmful to health. *BMJ* 2016;354:i4631 doi: doi: 10.1136/bmj.i4631

7 Entwisle DR, Astone NM. Some Practical Guidelines for Measuring Youth's Race/Ethnicity and Socioeconomic Status. *Child Dev* 1993; 65:1521-40.

8 Dahlgren G, Whitehead M. Policies and strategies to promote social equity in health. Institute for future studies. (revised version) 2007:14.

9 Galobardes B, Lynch JW, Smith GD. Is the association between childhood socioeconomic circumstances and cause-specific mortality established? Update of a systematic review. *J Epidemiol Community Health* 2008;62:387–90.

10 Hirsch D, Spencer N. Unhealthy lives; intergenerational links between child poverty and poor health in the UK, summary briefing. End Child Poverty Now Policy Briefing; 2008. http://www.donaldhirsch.com/unhealthylives.pdf.

11 Non Al, Román JC, Gross CL, Gilman SE, Loucks EB, Buka SL, et al. Early childhood social disadvantage is associated with poor health behaviours in adulthood. *Ann Hum Biol* 2016; 43(2): 144–153. doi:10.3109/03014460.2015.1136357.

For peer review only - http://bmjopen.bmj.com/site/about/guidelines.xhtml

12 Rajmil L, Sanmamed MJF, Choonara I, Faresjö T, Hjern A, Kozyrskyj AL, on Behalf of the International Network for Research in Inequalities in Child Health (INRICH). Impact of the 2008 Economic and Financial Crisis on Child Health: A Systematic Review. *Int J Env Res Public Health* 2014;11(6):6528–46.

13 Parmar D, Stavropoulou C, Ioannidis JPA. Health outcomes during the financial crisis in Europe: systematic literature review. *BMJ* 2016;354:i4588.

14 Taylor-Robinson D, Whitehead M, Barr B. Great leap backwards. *BMJ* 2014;349:g7350.

15 Rajmil L, Artazcoz L, García-Gómez P, Flores M, Hernández-Aguado I. Spanish children are most affected by the economic crisis, says the Spanish Society of Public Health (SESPAS). *BMJ* 2015;350:h1945.

16 Unicef Office of Research. Children of the recession: the impact of the economic crisis, on child well-being in rich countries; 2014. www.unicef-irc.org/publications/pdf/rc12-engweb.pdf.

17 Sdona E, Papamichail D, Ragkou E, Briana DD, Malamitsi-Puchner A, Panagiotopoulos T. Greek economic crisis and impaired perinatal parameters: experience from a public maternity hospital. *J Matern Fetal Neonatal Med* 2017;4:1-5. doi: 10.1080/14767058.2017.1342803.

18 Varea C, Terán LM, Bernis C, Bogin B, González-González A. Is the economic crisis affecting birth outcome in Spain? Evaluation of temporal trend in underweight at birth (2003–2012). *Ann Hum Biol* 2016;43(2):169–82 DOI: 10.3109/03014460.2015.1131847

19 Gunnlaugsson G. Child health in Iceland before and after the economic collapse in 2008. *Arch Dis Child* 2015;0:1–8. doi:10.1136/archdischild-2014-307196.

20 Cantillon B, Chzhen Y, Handa S, Nolan B. Children of Austerity. Impact of the Great Recession on Child Poverty in Rich Countries. UNICEF: Oxford University Press; 2017.

21 International Monetary Fund (IMF). Fiscal Monitor. Achieving More with Less. IMF: 2017. http://www.imf.org/en/Publications/FM/Issues/2017/04/06/fiscal-monitor-april-2017.

22 Krugman P. The case for cuts was a lie. Why does Britain still believe it? The austerity delusion. The Guardian: 29th April 2015. https://www.theguardian.com/business/ng-interactive/2015/apr/29/the-austerity-delusion

For peer review only

For peer review only - http://bmjopen.bmj.com/site/about/guidelines.xhtml

23 Ballinger G. Using generalized estimation equations for longitudinal data analysis. *Org Res Methods* 2004;7:127–50. doi:10.1177/1094428104263672.

24 French B, Heagerty PJ. Analysis of Longitudinal Data to Evaluate a Policy Change. *Stat Med*. 2008; 27(24): 5005–25.

25 Karanikolos M, Mladovsky P, Cylus J, Thomson S, Basu S, Stuckler D, et al. Financial crisis, austerity, and health in Europe. *Lancet* 2013;381:1323–31.

26 Eiríksdóttir VH, Ásgeirsdóttir TL, Bjarnadóttir RI, Kaestner R, Cnattingius S, Valdimarsdóttir UA. Low birth weight, small for gestational age and preterm births before and after the economic collapse in Iceland: a population based cohort study. *PLoS One* 2013;8:e80499. doi:10.1371/journal.pone.0080499.

27 Novoa AM, Ward J, Malmusi D, Díaz F, Darnell M, Trilla C, et al. Condicions de vida, habitatge i salut. Mostra de persones ateses per Càritas Diocesana de Barcelona. Barcelona; Càritas Diocesana de Barcelona; 2013.

28 Plataforma de afectados por la Hipoteca. Te quedarás en la Oscuridad. Desahucio, familia e infancia desde un enfoque de derechos. Madrid: PAH; 2016. http://pahmadrid.org/infancia/estudio-te-quedaras-en-la-oscuridad.

29 Rajmil L, Medina-Bustos A, Fernández de Sanmamed MJ, Mompart-Penina A. Impact of the economic crisis on children's health in Catalonia: a before–after approach. *BMJ* Open 2013; 3:e003286. doi:10.1136/bmjopen-2013-003286.

30 McKenna C, Law C, Pearce A. Increased household financial strain, the Great Recession and child health—findings from the UK Millennium Cohort Study. *BMJ Open* 2017;7:e015559. doi:10.1136/bmjopen-2016-015559

31 Marmot M. Social causes of the slowdown in health improvement. *J Epidemiol Community Health* 2018; doi: 10.1136/jech-2018-210580

32 Taylor-Robinson DC, Lai E, Bradshaw J, Wolfe I, Weeks A, Barr B, et al. Death rate continues to rise for poorest infants in England and Wales. *BMJ* 2018;360:k1090.

33 Taylor-Robinson D, Barr B. Death rate now rising in UK's poorest infants. *BMJ* 2017;357:j2258 doi: 10.1136/bmj.j2258.

For peer review only - http://bmjopen.bmj.com/site/about/guidelines.xhtml

Figure 1. Generalising Estimating Equation (GEE) model

GEE approach is an extension of Generalising linear models that provides a semi-parametric approach to longitudinal analysis of categorical and continuous measurements. In this study, a GEE model with link identity and normal distribution, and exchangeable working correlation structure was selected. Given y_{ij} *the response for country i at time-period j and* $\boldsymbol{x}_{ij}$ *the px1 vector of covariates, the marginal response* μ_{ij} *is estimated as:*

$$E(y_{ij}) = \mu_{ij} = \boldsymbol{x}'_{ij}\boldsymbol{\beta}$$

Where $\boldsymbol{\beta}$ *is a vector of regression coefficients. The (j,k) element of the working correlation matrix Ri is the estimated correlation between* y_{ij} *and* y_{ik}

$$Corr(y_{ij}, y_{ik}) = \begin{cases} 1 & j = k \\ \propto & j \neq k \end{cases}$$

81x60mm (300 x 300 DPI)

w only

Figure 2. Low birth weight

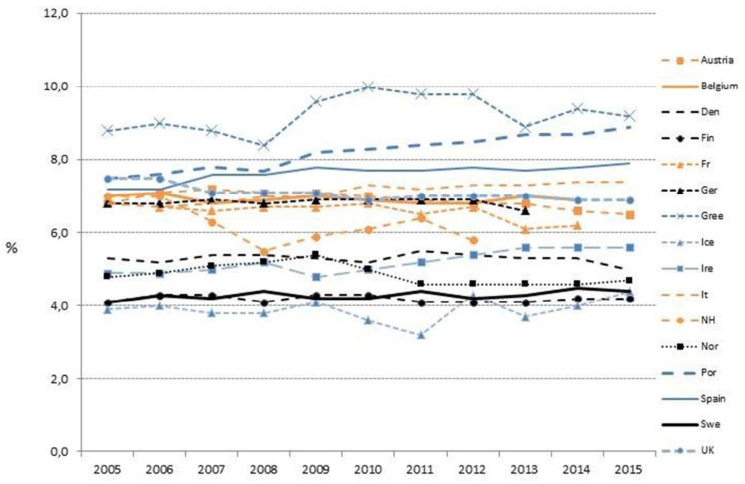

Low birth weight: live births weighing less than 2500 grs divided by the total number of live births

Source of data: Organisation for Economic Co-operation and Development (OECD) Family Database http://www.oecd.org/els/family/database.htm

High austerity level   Intermediate level   Low austerity level

81x60mm (300 x 300 DPI)

Figure 3. Infant mortality

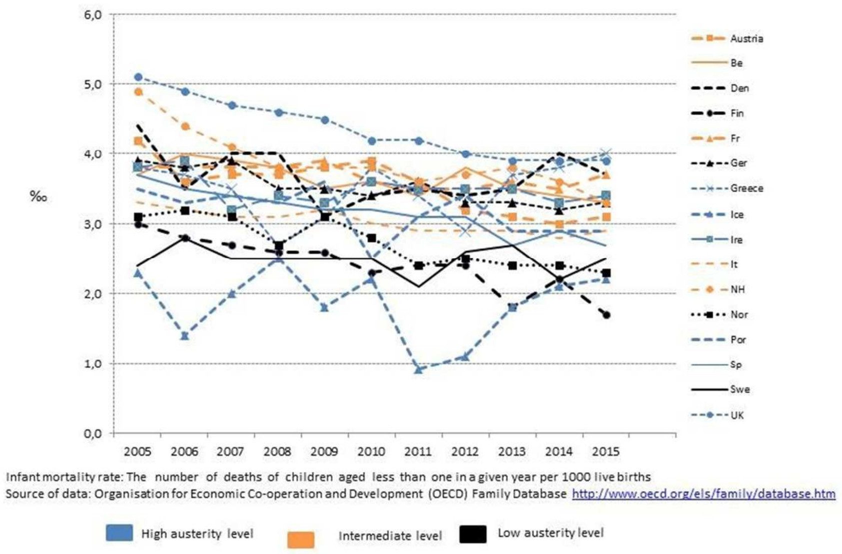

81x60mm (300 x 300 DPI)

w only

Figure 4. Child poverty rates

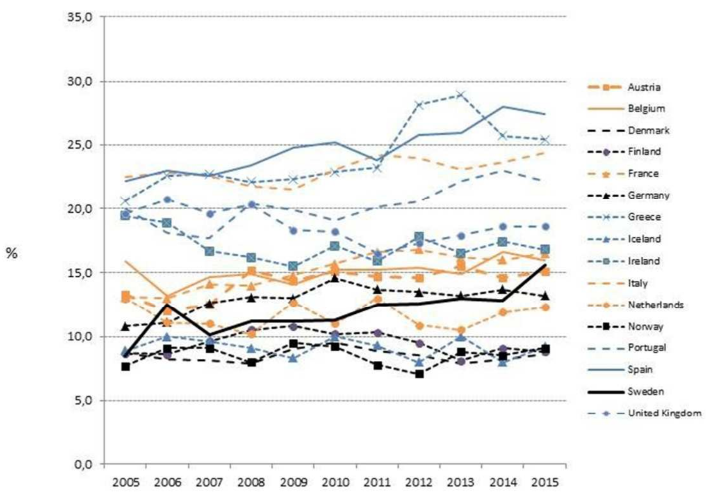

Child poverty rates: percentage of children living in households with income below 60% of the median.
Source of data: European Union-Survey of Income and Living conditions (EU-SILC)
http://ec.europa.eu/eurostat/web/microdata/european-union-statistics-on-income-and-living-conditions

High austerity level   Intermediate level   Low austerity level

81x60mm (300 x 300 DPI)

For peer review only

Figure 5. Severe material deprivation in families with primary education level

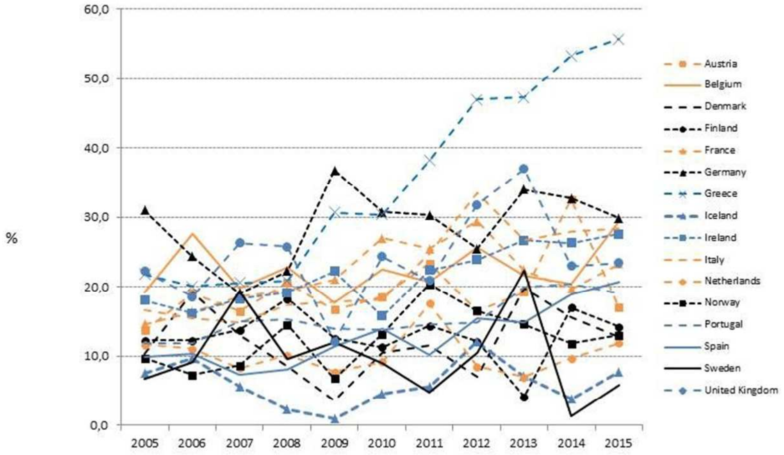

Severe material deprivation: proportion of children under 18 years of age living in families with primary level of education which cannot afford to pay for at least four out of the nine items considered basic.
Source of data: European Union-Survey of Income and Living conditions (EU-SILC)
http://ec.europa.eu/eurostat/web/microdata/european-union-statistics-on-income-and-living-conditions

High austerity level Intermediate level Low austerity level

81x60mm (300 x 300 DPI)

Figure 6. Pre-school investment

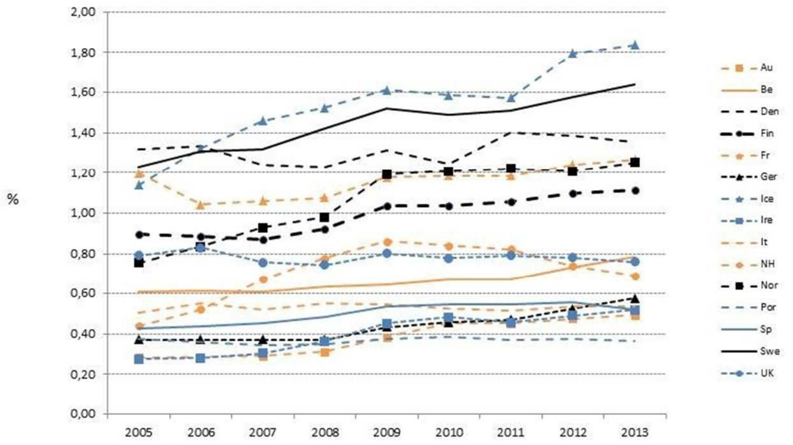

Pre-school investment (0-5 years old): annual percentage of Gross Domestic Product (GDP).
Source of data: Organisation for Economic Co-operation and Development (OECD) Family Database http://www.oecd.org/els/family/database.htm
No data available for Greece

High austerity level Intermediate level Low austerity level

81x60mm (300 x 300 DPI)

STROBE Statement—Checklist of items that should be included in reports of ***cross-sectional studies***

The present study is a longitudinal ecological study of trends in perinatal outcomes and social determinants of child health (SDCH) at country level. It was based on the analysis of routinely available data for the period 2005-2015 from sixteen European countries plus Australia and Canada. All the data and procedures were carried out following the data protection requirements of the European Parliament (Directive 95/46/EC of the European Parliament and of the Council of 24 October 1995 on the protection of individuals with regard to the processing of personal data and on the free movement of such data).

	Item No	Recommendation	Checklist (page No)
**Title and abstract**	1	(*a*) Indicate the study's design with a commonly used term in the title or the abstract	1
		(*b*) Provide in the abstract an informative and balanced summary of what was done and what was found	2
**Introduction**			
Background/rationale	2	Explain the scientific background and rationale for the investigation being reported	4
Objectives	3	State specific objectives, including any prespecified hypotheses	4 last para
**Methods**			
Study design	4	Present key elements of study design early in the paper	5
Setting	5	Describe the setting, locations, and relevant dates, including periods of recruitment, exposure, follow-up, and data collection	5
Participants	6	(*a*) Give the eligibility criteria, and the sources and methods of selection of participants	5
Variables	7	Clearly define all outcomes, exposures, predictors, potential confounders, and effect modifiers. Give diagnostic criteria, if applicable	5, 6
Data sources/ measurement	8*	For each variable of interest, give sources of data and details of methods of assessment (measurement). Describe comparability of assessment methods if there is more than one group	5, 6
Bias	9	Describe any efforts to address potential sources of bias	9, 2nd para
Study size	10	Explain how the study size was arrived at	5
Quantitative variables	11	Explain how quantitative variables were handled in the analyses. If applicable, describe which groupings were chosen and why	5, 6
Statistical methods	12	(*a*) Describe all statistical methods, including those used to control for confounding	6
		(*b*) Describe any methods used to examine subgroups and interactions	6
		(*c*) Explain how missing data were addressed	Not available for most of indicators included
		(*d*) If applicable, describe analytical methods taking account of sampling strategy	5, 6
		(*e*) Describe any sensitivity analyses	9
**Results**			
Participants	13*	(a) Report numbers of individuals at each stage of study—eg numbers potentially eligible, examined for eligibility, confirmed eligible, included in the study, completing follow-up, and analysed	Data from Eurostat and/or OECD was considered as representative at country level

For peer review only - http://bmjopen.bmj.com/site/about/guidelines.xhtml

		(b) Give reasons for non-participation at each stage	-
		(c) Consider use of a flow diagram	It was considered as not necessary
Descriptive data	14*	(a) Give characteristics of study participants (eg demographic, clinical, social) and information on exposures and potential confounders	5, 9
		(b) Indicate number of participants with missing data for each variable of interest	Not available for most of indicators included
Outcome data	15*	Report numbers of outcome events or summary measures	7
Main results	16	(*a*) Give unadjusted estimates and, if applicable, confounder-adjusted estimates and their precision (eg, 95% confidence interval). Make clear which confounders were adjusted for and why they were included	15, 16
		(*b*) Report category boundaries when continuous variables were categorized	6
		(*c*) If relevant, consider translating estimates of relative risk into absolute risk for a meaningful time period	It was not considered
Other analyses	17	Report other analyses done—eg analyses of subgroups and interactions, and sensitivity analyses	7
**Discussion**			
Key results	18	Summarise key results with reference to study objectives	2, 3, 10
Limitations	19	Discuss limitations of the study, taking into account sources of potential bias or imprecision. Discuss both direction and magnitude of any potential bias	9
Interpretation	20	Give a cautious overall interpretation of results considering objectives, limitations, multiplicity of analyses, results from similar studies, and other relevant evidence	9
Generalisability	21	Discuss the generalisability (external validity) of the study results	9
**Other information**			
Funding	22	Give the source of funding and the role of the funders for the present study and, if applicable, for the original study on which the present article is based	11

*Give information separately for exposed and unexposed groups.

Note: An Explanation and Elaboration article discusses each checklist item and gives methodological background and published examples of transparent reporting. The STROBE checklist is best used in conjunction with this article (freely available on the Web sites of PLoS Medicine at http://www.plosmedicine.org/, Annals of Internal Medicine at http://www.annals.org/, and Epidemiology at http://www.epidem.com/). Information on the STROBE Initiative is available at www.strobe-statement.org.

For peer review only

For peer review only - http://bmjopen.bmj.com/site/about/guidelines.xhtml
